# Inhibitory effect of LSOPC on AGEs formation and sensory quality in cookies

**DOI:** 10.3389/fnut.2022.1064188

**Published:** 2022-12-15

**Authors:** Qian Wu, Jiangying Tan, Jiabin Qin, Ziting Chen, Bing Li, Jianhua Xu, Weiting Jiao, Nianjie Feng

**Affiliations:** ^1^Key Laboratory of Fermentation Engineering (Ministry of Education), Cooperative Innovation Center of Industrial Fermentation (Ministry of Education & Hubei Province), Hubei Key Laboratory of Industrial Microbiology, National “111” Center for Cellular Regulation and Molecular Pharmaceutics, Hubei University of Technology, Wuhan, Hubei, China; ^2^Pinyuan (Suizhou) Modern Agriculture Development Co., Ltd., Suizhou, Hubei, China; ^3^State Key Laboratory of Tea Plant Biology and Utilization, Anhui Agricultural University, Hefei, Anhui, China

**Keywords:** advanced glycation end products, sensory quality, lotus seedpod oligomeric procyanidins, cookies system, inhibition

## Abstract

At the conclusion of the Maillard reaction (MR), free amino groups of proteins, amino acids, or lipids with the carboxyl groups of reducing sugars to form stable molecules known as advanced glycation end products (AGEs), which hasten aging and may potentially be the root cause of a number of chronic degenerative diseases. According to researches, lotus seedpod oligomeric procyanidins (LSOPC), a premium natural antioxidant produced from lotus waste, can be included in cookies to improve flavor and lower the risk of illnesses linked to AGEs. In this work, we used cookies without LSOPC as a control to examine the effects of adding various concentrations of LSOPC (0, 0.05, 0.1, 0.2, and 0.4%) on the AGEs formation and the sensory quality in cookies. The amounts of AGEs and N-ε-carboxymethyl lysine (CML) decreased with the increase of LSOPC concentration, indicating that the concentration of LSOPC was positively correlated with the ability to inhibit AGEs formation. It was also demonstrated that the amount of antioxidant capacity of the cookies increased significantly with the increase of LSOPC concentration. On the other hand, the chromaticity, texture, electronic nose, and other aspects of the cookies’ sensory attributes were also evaluated. The color of the cookies deepened and the flavor varied as LSOPC added content increased. The sensory quality of the cookies was examined, and the findings indicated that LSOPC would somewhat improve that quality. These findings implied that AGEs formation could be decreased in cookies while also enhancing their sensory quality by adding LSOPC.

## 1 Introduction

A non-enzymatic browning reaction known as Maillard Reaction (MR), or carbonyl ammonium reaction, is a common occurrence in food processing and storage (1). At room temperature or when heated, carbonyl (such as reducing sugars) and amino (such as amino acids, peptides, proteins, etc.) compounds can undergo a series of oxidation, cyclization, dehydration, and polymerization reactions that produce a variety of Maillard reaction products (MRPs), which also affect the color and aroma of foods (2).

When food is processed, dietary protein glycation can lead to the development of potentially dangerous chemicals like advanced glycation end products (AGEs) (3). The advanced glycation end products (AGEs), which are classified as exogenous (found in food) and endogenous (found *in vivo*), are produced through MR and other routes (4). A significant source of AGEs in the body is dietary intake of AGEs (5). According to estimates, only 1/3 of AGEs consumed through diet enter the bloodstream and are then eliminated from the body through the kidneys, leaving the other 2/3 to remain in the body (6). Complex cross connections between AGEs and functional proteins may arise in the body as AGEs build up, altering the structural makeup of proteins. Their biochemical processes are subsequently altered as a result of this event, resulting in diabetes problems and other health issues (7).

Numerous studies have demonstrated that natural flavonoids have antioxidant properties and can inhibit the synthesis of Schiff bases to lessen the generation of AGEs (8). Due to its antioxidant capabilities, the favonoids class known as lotus seedpod oligomeric procyanidins (LSOPC) is the subject of the most research (9, 10). LSOPC, a sort of procyanidin that was taken from the mature lotus housing receptacle, has recently been proven to have anti-glycation potential (11, 12). LSOPC is biologically active in a variety of ways, including antioxidant, free radical scavenging, anticancer, cardiovascular protective, and cell proliferative properties, according to recent studies (13, 14). They can also effectively eliminate different reactive oxygen species (ROS). On the other side, it can bind to active carbonyl compounds to stop the AGEs formation from being produced (15).

**SCHEME 1:**
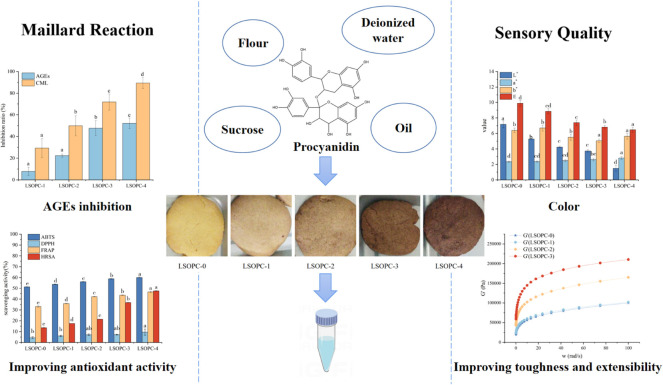
Effect of LSOPC on cookies.

In light of its chemical composition and physical surroundings, cookie is a complicated system that is full of fats, proteins and carbohydrates. Some studies showed that the GO content was 338-1936 μg/100 g and the MGO content was 727-1,397 μg/100 g in cookies (16). Uncertainty exists regarding how LSOPC affects AGEs formation and the subsequent effects on cookies quality. In this research, LSOPC, as a cookies additive and food function factor, was discovered to prevent AGEs formation. Investigations were also done on how LSOPC affected the physicochemical makeup and flavor of cookies (Scheme 1).

## 2 Materials and methods

### 2.1 Materials

Milk was obtained from Weidendorf (Shanghai, China). Sugar and low-gluten wheat flour were bought from Angel Yeast Co., Ltd. (Yichang, China). Eggs, sodium bicarbonate food grade and ammonium bicarboantewere food grade were purchased from a local supermarket. Standard N-ε-carboxymethyl lysine (CML) was purchased from Toronto Research Chemicals (Toronto, Canada). DPPH (2,2-diphenyl-1-picrylhydrazyl) and ABTS [2,2’-azinobis (3-ethylbenzothiazoline-6-sulphonic acid)] were brought from Sinopharm Chemical Reagent Factory (Shanghai, China). Sodium acetate, 2,4,6-tripyridyl-s-triazine (TPTZ), ferric chloride and ethanol were purchased from Sigma-Aldrich (St Louis, MO). Solvents and chemicals such as methanol (HPLC grade), chloroform (HPLC grade) and sodium borohydride solution (pH 13-14) were supplied by Fisher Scientific (Fairlawn, NJ, USA). All chemicals used were of analytical grade unless otherwise stated.

### 2.2 Prepare of cookies

The model cookies were produced as Laura Roma ’n al. Summary (17) with some modifications. Procyanidin was uniformly incorporated into 80 g of soft flour at different levels (0, 0.05, 0.10, 0.20, and 0.40%). Sucrose (35 g), oil (32 g), salt (1 g), sodium bicarbonate (0.8 g), ammonium bicarbonate (0.4 g), deionized water (17.6 g) and procyanidin-enriched flour were well mixed to form bread dough. After thoroughly mixing the ingredients, the dough was flattened out to create 5 cm in diameter by 2 mm thick discs. Following that, samples were baked for 15 minutes at 180°C. Cookies models were identified as LSOPC-0 (without addition of procyanidin compounds), and LSOPC-1 (0.05% LSOPC), LSOPC-2 (0.1% LSOPC), LSOPC-3 (0.2% LSOPC), LSOPC-4 (0.4% LSOPC), respectively. A mixture of ground cookies (0.2 g) and water (10 mL) were ultrasonically shocked for an hour before being centrifuged at 25°C for five minutes at a speed of 3000 rpm to obtain liquid for sample extraction.

### 2.3 Free fluorescent AGEs determination

The procedure was based on previous methods by X. Zhang et al. (18) with minor modifications. Fluorescence spectrophotometry was used to measure the formation of AGEs in a 96-well microplate using 370 ± 40 nm and 440 ± 40 nm as the excitation and emission wavelengths, respectively. Formation of fluorescent AGEs was estimated by analyzing the fluorescence in samples. The sample without LSOPC served as the control group. The inhibition was calculated as:


$$\mathrm{Inhibition}(\%)=\frac{{\text{A}}_{\text{control}}-{\text{A}}_{\text{sample}}}{{\text{A}}_{\text{control}}}\times 100$$

A_sample_ and A_control_ were the absorbance of the sample and the control, respectively.

### 2.4 N-ε-carboxymethyl lysine (CML) determination

The CML of cookies samples were extracted according to Drusch, Faist, and Gengjun Chen et al. (19) with some modification. Each sample (0.50 g) was mixed with 2 ml sodium borohydride (pH 13, prepared with 0.1 M NaOH) reducing agent and left at 4°C for 10 h. The supernatant after centrifugation was filtered through the preactivated solid phase extraction PCX column. Before HPLC-MS2 analysis, the fifinal CML extracts were concentrated until dry and dissolved in 1 ml of 0.1% formic acid, followed by a membrane fifiltration (nylon, 0.22 μm). Then 15 μl samples were injected into an Eclipse Plus C_18_ column (2.1 × 50 mm, 5 μm, Agilent Technologies, Germany) with 0.2% formic acid (solvent A) and acetonitrile (solvent B) as mobile phases at 30°C. The chromatography run time was set to 25 min and the flow rate was 0.2 mL/min. The gradient program was as follows: 0-0.5 min, 90% A; 0.5-4.0 min, 90%-60% A; and 4.0-25.0 min, 60% A. The positive ion mode was used to operate the mass spectrometer with multiple reaction monitoring. The capillary voltage was held at 4 kV and the nitrogen temperature was maintained at 300°C. For the quantitative and qualitative analyses of CML (*m/z* 205), respectively, the fragments at *m/z* 84 and 130 were employed with MassHunter Data and MassHunter Qualitative (Agilent Technologies, Germany).

### 2.5 Determination of total phenol content (TPC)

The phenolic content of cookies was determined by Folin-phenol method as described by Ervina (20). The absorbance of the mixture was read at 765 nm using microplate reader. The total phenolic content of the sample was calculated using a standard curve plotting the amount of gallic acid (μg) against the absorbance at 765 nm.

### 2.6 LSOPC degradation analysis

The concentration of LSOPC was measured using a UV spectrometer at 546 nm (18). Standard curves were prepared using standard compounds under the same conditions. The degradation was calculated as:


$$\mathrm{Degradation}(\%)=\frac{{\text{C}}_{\text{a}}-{\text{C}}_{\text{b}}}{{\text{C}}_{\text{b}}}\times 100$$

C_a_ and C_b_ were the concentration of LSOPC before and after treatment, respectively.

### 2.7 Antioxidant activity analysis

#### 2.7.1 ABTS radical scavenging assay

For ABTS assay, the procedure followed the method of Kriengsak Thaipong et al. (21) with some modifications. A 7.4 mM aqueous solution of ABTS and 2.6 mM K_2_O_8_S_2_ (final concentration) combination was used to create an ABTS^+^ stock solution, which was then left to stand for 12 to 16 hours at room temperature with no light. The stock solution was diluted in water/ethanol (50:50, v/v) on the day of the analysis to create an ABTS^+^ working solution. The working solution of ABTS^+^ had an absorbance of 0.70 ± 0.02 AU at 734 nm. The ABTS radical scavenging activity was calculated using the following formula:


$$\mathrm{ABTS}\mathrm{radical}\mathrm{scavenging}\mathrm{activity}(\%)=\frac{{\text{A}}_{\text{control}}-{\text{A}}_{\text{sample}}}{{\text{A}}_{\text{control}}}\times 100$$

Where A_control_ was the absorbance of ABTS and A_sample_ was the absorbance of the sample and ABTS^+^ at 734 nm.

#### 2.7.2 DPPH radical scavenging assay

The free radical scavenging capacity of each cookies extract were estimated according to the DPPH assay by Singh et al. (22) with some modifications. A working solution of DPPH reagent with a final concentration of 1 mM was made in 50% ethanol. The DPPH antioxidant activity of each sample was calculated using the following formula:


$$\mathrm{DPPH}\mathrm{radical}\mathrm{scavenging}\mathrm{activity}(\%)=1-\frac{\left(A-{\text{A}}_{\text{b}}\right)}{{\text{A}}_{0}}\times 100$$

Where A_0_ was the absorbance of DPPH, A was the absorbance of the sample and DPPH, and A_b_ was the absorbance of the sample at 517 nm.

#### 2.7.3 2,2′-azinobis-(3-ethyl-benzothiazoline-6-sulfonic) acid (FRAP) assay

The ferric-reducing power was tested using the assay of Ilkay Erdogan-Orhan et al. (23) with some modifications. A ferric complex (Fe^3+^-TPTZ) is reduced to the ferrous form (Fe^2+^-TPTZ) in the presence of antioxidants in this procedure. The FRAP reagent was obtained by mixing 10 ml sodium acetate buffer (pH 3.6, 300 mM), 1 mL TPTZ (10 mM) and 1 mL FeCl_3_⋅6H_2_O solution (20 mM). A reaction mixture consisting of 10 μl of the extract and 300 μl of FRAP reagent was incubated at 37°C for 4 min. Using the microplate reader mentioned above, the absorbance was measured at 593 nm. The Ferric reducing antioxidant power was calculated as followed:


$$\mathrm{Ferricreducing}\mathrm{antioxidant}\mathrm{power}(\%)=\frac{A_{\text{sample}}-A_{\text{control}}}{{\text{A}}_{\text{black}}}\times 100$$

While A_sample_ was the absorbance of the sample and FRAP at 593 nm, A_control_ was the absorbance of FRAP at 593 nm, A_black_ was the absorbance of the sample at 593 nm.

#### 2.7.4 Hydroxyl radical scavenging ability (HRSA) in cookies

The free radical scavenging capacity of each cookies extract were estimated according to the HRSA assay by Li (24) with some modifications. Test tubes containing 1 mL of sample, ferric sulfate, and a solution of salicylic acid and ethanol were then filled with 1 ml of hydrogen peroxide, water, and allowed to stand in a 37°C water bath for 30 min. The absorbance was calculated at 510 nm using distilled water as a blank as a reference. The same procedure was used to calculate the comparable absorbance when distilled water was used in place of hydrogen peroxide. Using the following formula, the hydroxyl radical scavenging rate was expressed as %:


$$\mathrm{Hydroxyl}\mathrm{radical}\mathrm{scavenging}\mathrm{rate}(\%)=1-\frac{A_{\text{sample}}-A_{\text{control}}}{A_{\text{black}}}\times 100$$

Where A_sample_, A_control_ and A_black_ were the absorbance of the sample, control and blank groups at 510 nm, respectively.

### 2.8 Determination of water

#### 2.8.1 Determination of moisture content

The amount of moisture in the samples was measured using the moisture meter set to 105°C (25).

#### 2.8.2 Determination of water activity

The amount of Aw in the samples was measured using the AquaLAB CX-2 (Decagon Devices Inc., Pullman, WA) set to 25°C.

#### 2.8.3 Nuclear magnetic resonance

The cookies were removed and transferred to the NMR tube, and the water distribution was measured by nuclear magnetic resonance imaging analyzer. The following test parameters were used: 8 repeated scans, 24 sample points, and a relaxation decay time of 2,000 ms (26). The CPMG pulse sequence was used to calculate the relaxation time T_2_.

### 2.9 Determination of pH

The sample (0.1 g) was mixed with distilled water (10 ml) and ultrasonically shocked for 3 min (37°C). The portable pH meter was used to measure the pH of supernatant after the mixture was centrifuged at 25°C and 3000 rpm.

### 2.10 Determination of color

Cookies chroma were measured according to the method of Siti Rashima (27). Color of cookies were measured with a Konika Minolta reflectance spectrophotometer CM-3500d (Konica Minolta Sensing INC, Osaka, Japan), and readings were expressed as L* (lightness), a* (redness) and b* (yellowness) values. For various areas of the cookies center, independent detections were made six times and averaged. The value E was calculated according to the equation:


$$\Delta E=\sqrt{\left(\Delta L^{*}2+\Delta a^{*}2+\Delta b^{*}2\right)}$$

### 2.11 Instrumental texture analysis

The texture properties of cookies were measured according to the method of Fatma Bouaziz (28). The samples’ textures were evaluated using the texture analyzer with a P/32 cylindrical column probe at 30.0 g for the trigger force and 2.0 mm/s for the pretest, test, and post-test speeds. The final results were examined using the TPA software.

### 2.12 Electronic-nose data acquisition

The PEN 3 E-nose had ten distinct metal oxide sensors to target various fragrance compounds. Before being measured, samples (1.0 g) and 10% saline were balanced in a 37°C water bath for 30 min. The test conditions were set as follows: flow rate of carrier gas was 300 ml/min, pre-injection time was 5 s, sample measurement time was 200 s, reset time was 5 s and cleaning time was 120 s. The data were analyzed by principal component analysis (PCA) using the E-nose software system (29).

### 2.13 Gas chromatography-mass spectrometry (GC-MS) analysis

The GS in several LSOPC cookies samples were measured using an altered extraction technique (30). To extract the taste components, a commercial solid-phase microextraction fiber with an 85 μm carbowax/polydimethylsiloxane coating was inserted into a manual holder and placed into the headspace above the sample for 30 min at 60°C (31). The fiber was inserted into the needle and injected into the GC-MS system for desorption at 250°C for 5 min.

Gas chromatography-mass spectrometry analysis was then carried out on an Agilent 6890 GC system coupled to an Agilent 5,975 inter quadrupole mass spectrometer. The GC separation was conducted on an HP-5MS capillary column (30 m × 0.25 mm, 0.25 μm). Helium was used as carrier gas at the rate of 1 ml/min. The initial oven temperature was set at 40°C (held for 2 min), raised to 160°C at 4°C/min and then programmed heating to 250°C at 10°C/min (held for 2 min). The mass spectrometer condition was: the ionization energy: 70 eV, the scan range: *m/z* 30-500, the ionization source temperature: 230°C, and the quadrupole temperature: 150°C.

### 2.14 Determination of the rheological propriety

The rheological properties of sample were studied by a DHR-3 rotary rheometer (TA Instruments Inc., USA) at 25°C. The freshly prepared samples were measured the linear viscoelastic region by dynamic strain sweep. Then, with the plate diameter set at 40 mm, the gap at 1 mm, the temperature at 25°C, the scanning strain set at 1%, and the frequency at 0.1-10 Hz, the frequency curves of the storage modulus (G’) and loss modulus (G”) were produced. The identical rheological system and circumstances were used to duplicate all samples (32).

### 2.15 Statistical analysis

All tests and analyses were performed in triplicate. Data were analyzed using SPSS 21.0 (expressed as mean ± SD), and differences were considered statistically significant at *p* < 0.05. Graphs were created using Origin 2021.

## 3 Results and discussion

### 3.1 The effect of LSOPC on fluorescent AGEs and CML formation

In the initial stages of non-enzymatic protein glycation, interactions between reducing sugars and free amino groups result in the formation of Schiff bases and Amadori groups, which in turn lead to the formation of AGEs (33). Fluorescence, brown hue, and cross-linking are characteristics of AGEs development (34). CML is a significant example of non-fluorescent AGEs. On the one hand, CML can bind to specific receptors and alter protein and cellular functions through physiological responses, causing pathological changes in the organism. On the other hand, CML can also cross-link with proteins and alter the normal function of some matrix protein molecules (35). Then, fluorescent AGEs and CML inhibition percentage were analyzed in the different cookies’ formulations.

**FIGURE 1:**
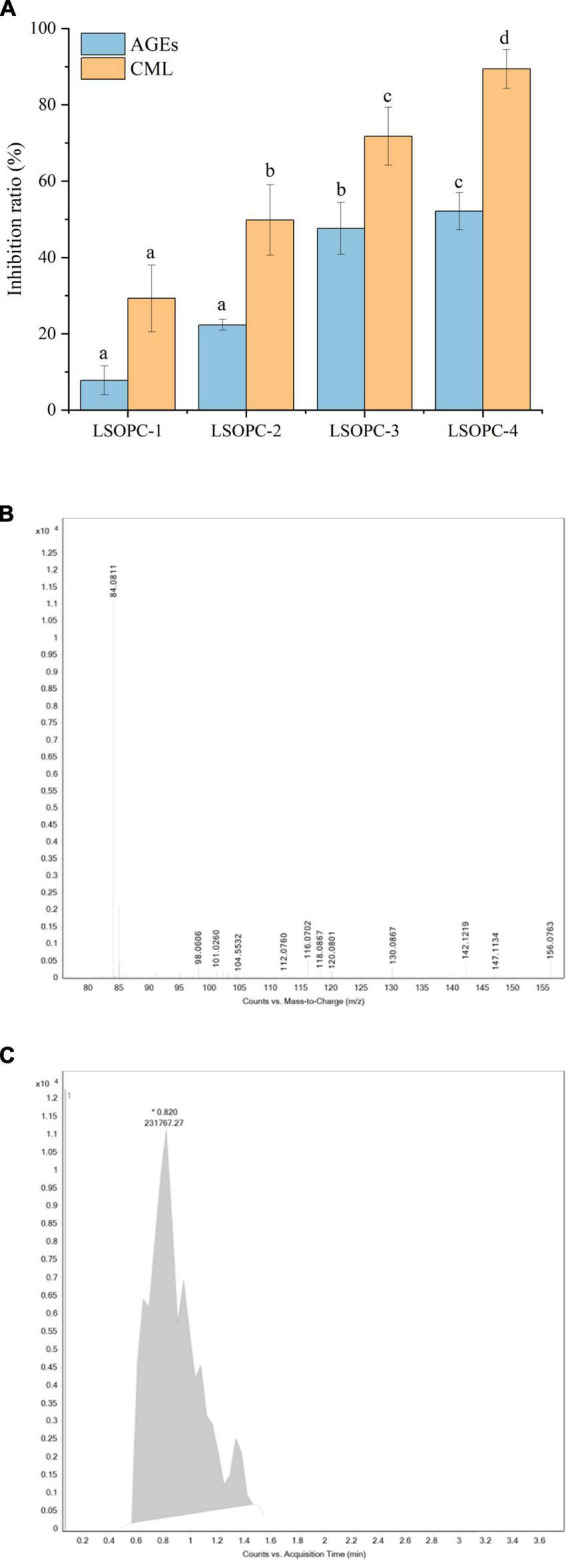
Effect of different concentrations of LSOPC on inhibition rate of AGEs and CML in cookies **(A)**. Ion spectra of the standard substance CML **(B)**. Peak area of the standard substance CML **(C)**. Significant differences (*p* < 0.05) of data values were indicated by different letters.

The inhibition rates of fluorescent AGEs and CML were favorably linked with the concentration of LSOPC, as shown in Figure 1A, when cookies at various LSOPC concentrations were applied. The steady increase in inhibitory of fluorescence AGEs formation in the LSOPC-treated samples suggested that LSOPC might prevent fluorescence AGEs from accumulating during the baking process. Following were the inhibition rates of AGEs at various LSOPC addition concentrations: LSOPC-1 (7.80%, *p* < 0.05), LSOPC-2 (22.35%, *p* < 0.05), LSOPC-3 (47.62%, *p* < 0.05) and LSOPC-4(52.12%, *p* < 0.05). These findings demonstrated that the four LSOPC cookies sample concentrations significantly inhibited the production of AGEs, particularly when LSOPC was added at the concentration of 0.4%. Dicarbonyl groups are reactive intermediates in the oxidative breakdown of Schiff bases and are precursors of AGEs. Reduced oxidative stress and the subsequent molecular alterations brought on by these precursors are made possible by antioxidants with free radical scavenging capability. Additionally, numerous studies revealed that one of the primary ways polyphenols stop the development of AGEs is by blocking dicarbonyl molecules (36).

The primary ion fragment at *m/z* 84, which was depicted in the secondary mass spectra of Figure 1B, can be used to determine the presence of definite CML in the LSOPC-0 cookies sample, and the extracted ion flow chromatograms, which are depicted in Figure 1C, can be used to calculate the CML peak area in the sample. To examine their inhibitory effects on AGEs formation, four concentrations of LSOPC were added to cookies in the current investigation. Each sample’s CML content was extracted, measured by HPLC. The addition of natural plants or plant extracts decreased the formation of AGEs in food or simulated systems, according to a number of studies (19). In this experiment, LSOPC is a natural plant extract, while CML is AGEs lacking UV absorption and fluorescence characteristics. The outcome matched the values that were reported by a number of investigations (37), which revealed that the addition of LSOPC decreased the quantity of CML in the cookies system.

### 3.2 The effect of LSOPC on total phenol content (TPC) and LSOPC degradation in cookies

**FIGURE 2:**
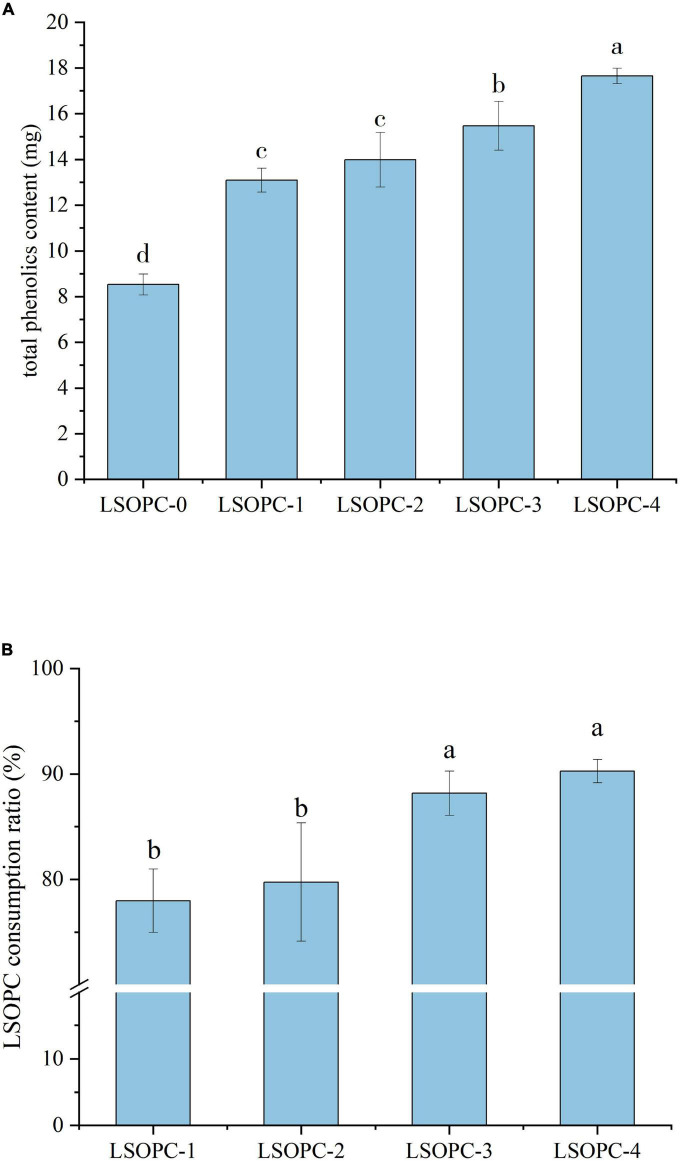
Effect of different concentrations of LSOPC on total phenol content in cookies **(A)**. Effects of different concentrations of LSOPC on its consumption ratio in cookies **(B)**. Significant differences (*p* < 0.05) of data values are indicated by different letters.

Certain chemicals or variables in the system were linked to an increase in AGEs inhibition and a decrease in CML content. In the cookies samples with varying concentrations of LSOPC, as shown in Figure 2A, the total phenolic content and LSOPC consumption rate both showed a rising trend with increasing LSOPC concentration (*p* < 0.05). Numerous studies had demonstrated that LSOPC includes a wide variety of phenolics and flavonoids (38). The overall phenolic content of the entire system would therefore rise as a result of the inclusion of LSOPC. The samples of cookies ranged in total phenolic content from 0.09 mg/g to 0.18 mg/g. Additionally, the LSOPC-0 sample’s total phenolic content was not zero even without the addition of LSOPC, indicating that the cookies system itself includes some total phenols in addition to the LSOPC (39). As a result, it’s possible that the overall phenol content of the cookies system itself also made a little contribution to the prevention of AGEs and CML formation.

As shown in Figure 2B, the LSOPC degradation rates of the LSOPC-1, LSOPC-2, LSOPC-3, and LSOPC-4 sample groups all exceeded 75% when LSOPC-0 was used as a reference. The great majority of polyphenols in the cookies system interact with proteins and have some impact on the cookies system, according to this. LSOPC, however, is also greatly influenced by temperature. The LSOPC consumption ratio might likewise decrease as heating duration varied (40). Combining Figure 2A, it can be shown that the amount of LSOPC added, the total phenolic content, and the rate of LSOPC consumption all affected how effectively the cookies system inhibited the formation of AGEs. LSOPC that interacted with the MR and hindered the oxidation reaction in the MR steadily increased as LSOPC concentration rose. Therefore, the more strongly the production of AGEs and CML, the MR’s products, was inhibited.

### 3.3 Antioxidation ability

**FIGURE 3:**
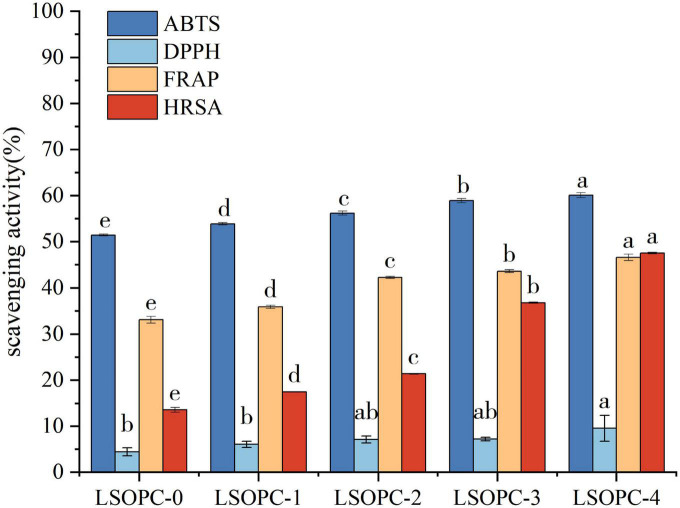
Effect of LSOPC on ABTs, DPPH, HRSA, FRAP scavenging activity. Significant differences (*p* < 0.05) of data values were indicated by different letters.

LSOPC might have prevented AGEs formation from forming in the system by improving a function of the cookies system. Numerous *in vitro* and *in vivo* studies had provided evidence that oxidative stress and carbonyl stress were intimately associated to the formation of AGEs (11). The utilization of four methods to measure antioxidant activity (ABTS, DPPH, FRAP, and HRSA) might give researchers a better knowledge of the various anti-radical properties of varied LSOPC cookies concentrations. Results for total antioxidant activity were shown in Figure 3.

When extra amounts varied in the samples of LSOPC cookies, DPPH, ABTS, FRAP, and HRSA largely showed a growing trend (*p* < 0.05), as shown in Figure 3. ABTS, FRAP, and HRSA all demonstrated a marked improvement in their capacity to scavenge free radicals with an increase in LSOPC content (*p* < 0.05). When compared to LSOPC-0 cookies, LSOPC-4 cookies exhibited a significantly better ability to scavenge DPPH radicals.

In conclusion, the LSOPC-4 sample group had the greatest matching total phenolic content and the maximum antioxidant capacity. This suggested that there was a connection between total phenolic content and antioxidant ability, as this experimental work found a positive association between them (41). This was in line with the findings of earlier studies (42): the polyphenol content was positively linked with the rate at which DPPH radicals were scavenged in the samples, and the correlation was very significant. The ability of polyphenolic compounds to scavenge free radicals was still quite strong, notwithstanding the possibility that they might interacted with protein digestion to create peptides. Therefore, the presence of polyphenolic compounds helped the cookies system’s antioxidant capacity to some level.

Additionally, the antioxidant capacity had a direct impact on the oxidation processes occurring in the MR. According to the experimental concentration range, the higher the concentration of LSOPC, the greater its antioxidant capacity, and consequently, the greater its ability to inhibit oxidation reactions in the MR and the generation of AGEs, i.e., the higher the AGEs inhibition rate, which was also consistent with the aforementioned experimental findings. Therefore, it was possible that phenolic compounds’ antioxidant activity to enhance the antioxidant properties of foods might play a role in LSOPC’s suppression of AGEs production.

### 3.4 Water

#### 3.4.1 Moisture content and water activity

Water activity and water content were both potential variables influencing how AGEs react. Therefore, it was determined how adding LSOPC would affect the moisture level and water activity in the cookies system.

As shown in Table 1, the water activity was in the range of 0.63-0.66 and the moisture was in the range of 9.30-9.57% as different amounts of LSOPC were added, but no discernible differences were identified when the results were compared. This demonstrates that, within the experimental concentration range, LSOPC had no impact on the moisture content and water activity of the cookies sample. This was consistent with the conclusions of Navarro et al. (43).

**TABLE 1:** The effect of LSOPC on the color and skin hardness of the cookies system (*p* < 0.05).

**Sample**	**LSOPC concentration (mg)**	**Skin hardness**	**Moisture content (%)**	**Water activity**
LSOPC-0	0	3008.872 ± 248.007^a^	9.3600 ± 0.33^a^	0.6290 ± 0.009^a^
LSOPC-1	0.5	2295.191 ± 88.331^b^	9.5733 ± 0.38^a^	0.6473 ± 0.005^a^
LSOPC-2	1.0	1808.606 ± 157.347^c^	9.2967 ± 1.96^a^	0.6540 ± 0.024^a^
LSOPC-3	2.0	2649.493 ± 342.066^ab^	9.3267 ± 0.91^a^	0.6583 ± 0.018^a^
LSOPC-4	4.0	2323.119 ± 180.902^b^	9.3667 ± 0.25^a^	0.6597 ± 0.013^a^

The data were given as mean ± S.D. (*n* = 3).

^a−c^Different letters indicated a significant difference (*p* < 0.05).

#### 3.4.2 Low-field NMR properties

The MR was also impacted by the type of water in the food system (44). The LF-NMR measurement was carried out to assess the mobility and distribution of water in all samples.

**FIGURE 4:**
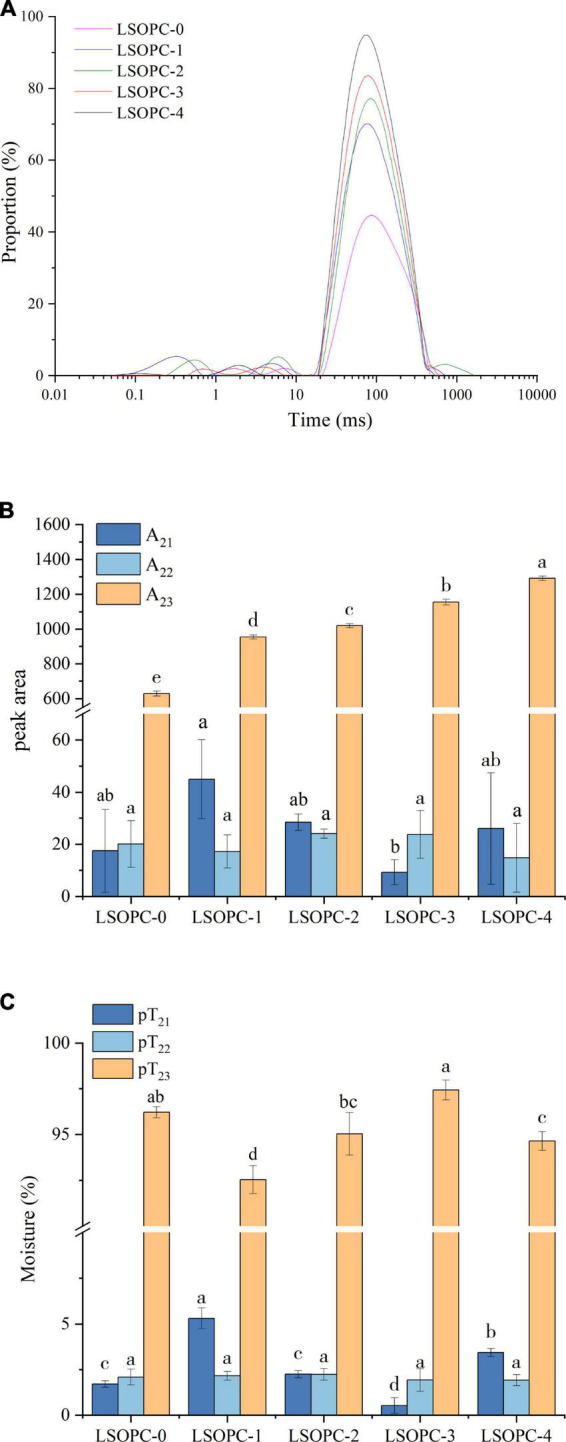
Effect of LSOPC on the relaxion time in cookies **(A)**. Effect of LSOPC on the peak area of different water in cookies **(B)**. Effect of LSOPC on the content of different water in cookies **(C)**. Significant differences (*p* < 0.05) of data values are indicated by different letters.

Typically, a dispersed exponential with one to three separate peaks could be used to characterize the NMR signal reduction. Figure 4A showed distributions of T_2_ relaxation times measured on the LSOPC cookies samples system. There were three kinds ways that water was distributed in cookies, 0.1 ms < T_21_ < 1 ms, 1 ms < T_22_ < 10 ms, 10 ms < T_23_ < 1,000 ms (26, 36). The water found in macromolecular structures (T_21_), the water that was more mobile and imprisoned in the protein-dense network (T_22_), and the loose water in the extra-protein network space (T_23_) were individually investigated as components and populations (45, 46).

From Figure 4A, the fluidity of water increased when it transitioned from a low relaxation time to a high relaxation time. The water migrated from low to high relaxation times with the addition of various concentrations of LSOPC, indicating that throughout the reaction, macromolecules like the proteins in the cookies established fewer interactions with water and the water became more mobile.

According to Figures 4B, C, the amount of loose water in the extra-protein network space (T_23_) varied the most among the cookies sample groups while the relaxation time of water found in macromolecules (T_21_) and water that was more mobile and trapped within protein-dense networks (T_22_) showed no trend change as the concentration of LSOPC addition increased. The LSOPC-4 sample had the highest content of loose water in the extra-protein network space (T_23_), which suggested that this sample lost less loose water than other samples did in this area. This might be because there were less hydrogen bonds created between large molecules, like the proteins in cookies, when LSOPC was present, making it simpler for water to bind with small-molecule polyphenols.

The aforementioned experimental results demonstrated that although the addition of LSOPC had no effect on the total moisture content and water activity, it might still have an impact on the reaction by altering the moisture distribution state of the cookies system, which would have an impact on the formation of AGEs.

### 3.5 pH properties

It was particularly well known that pH had a significant impact pace of the MR. The buffering ability of the system and the initial pH of the reagents influenced both the velocity and the orientation of the course way of the MR. When the pH was acidic, the Maillard reaction was thought to occur at a modest rate; however, when the pH rose, the rate increases until it reached a maximum at pH 10. The rate-determining factor at high pH values was a deficiency of H^+^ ions, which were necessary to catalyze both the Amadori and Heyns rearrangements.

**FIGURE 5:**
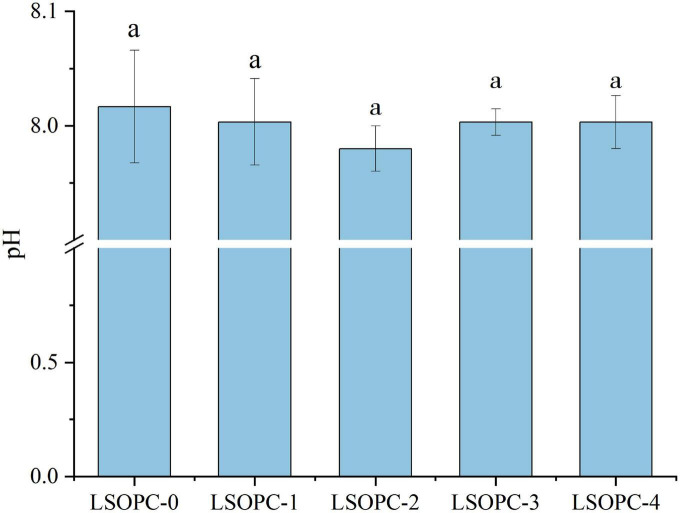
Effects of different concentrations of LSOPC on pH in cookies. Significant differences (*p* < 0.05) of data values were indicated by different letters.

As the MR moves on, the pH of the system falls as short chain acids were simultaneously formed and basic amino groups are consumed (47). However, the pH of cookies with various amounts of LSOPC had no statistically significant difference (*p* > 0.05), as shown in Figure 5, ranging between 7.98 and 8.02, which was compatible with the research findings of Navarro M et al. (43). This proved that, due to the effect of the LSOPC’s addition on potentially suppressing MR, the same system with varying LSOPC concentrations could maintain identical pH values.

### 3.6 Color properties

**FIGURE 6:**
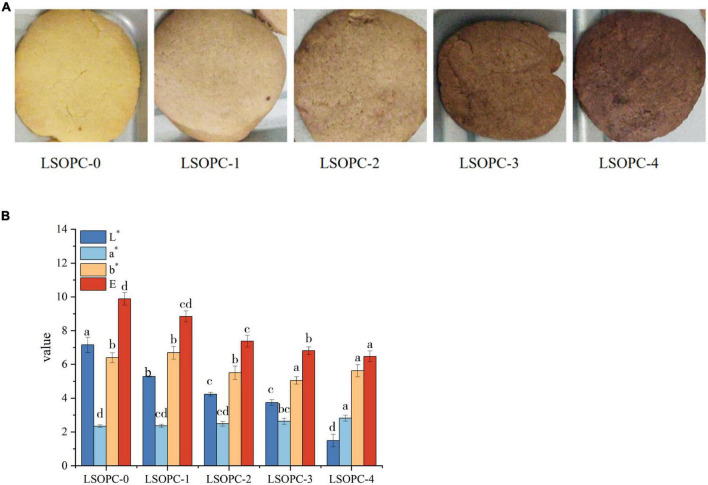
Original images of cookies **(A)**. Chromatic value of cookies **(B)**. Significant differences (*p* < 0.05) of data values were indicated by different letters.

The color of the cookies was impacted by the inclusion of phenolic chemicals, as seen in Figure 6A. LSOPC-0 was significantly lighter than LSOPC-1, LSOPC-2, LSOPC-3, and LSOPC-4. The LSOPC-4 sample attained the maximum level of color variation, as seen in Table 1. The color of the LSOPC itself had a significant impact on this color fluctuation.

The results were made more objective by using a colorimeter to measure the color brightness of the cookies in order to evaluate their sensory quality. Positive a* denotes redness, and the bigger the a*, the redder the cookies. Positive b* showed yellowness, and the greater the b*, the more yellow the cookies. L* indicated how white the cookies were; the greater the L*, the whiter the cookies.

The outcomes were displayed in Figure 6B. The L* value of the cookies’ surface reduced as the LSOPC content rose, but the a* value slightly increased. Next, the cookies’ whiteness and redness both marginally changed as the LSOPC level rose. As the LSOPC content of the cookies system grew, it was hypothesized that the color of the cookies might be more influenced by the color of the LSOPC itself. Additionally, the browning byproducts of the MR might influence the color of the cookies skin (18). The synergistic interaction of these two elements might account for the drop in L* value and the rise in a* value. A drop in whiteness and a proportional difference in redness resulted from an increase in LSOPC concentration because the color of LSOPC itself had a stronger impact. On the other hand, pan-yellowing went through a period of development. In the range of 0-0.5 mg/g, the b* value increased with increasing LSOPC content, but when the concentration is 0.5 mg/g – 4 mg/g, there was a certain decrease, i.e., the yellowness decreased in a certain concentration range. Because LSOPC had not yet completely suppressed the formation of AGEs in the prior concentration range, where the color of browning products dominated (48), and the pan-yellowness was raised, the increase in b* in the range of 0-0.5 mg/g might be the result. Beyond this point, however, LSOPC would take center stage and the yellowness will diminish. Additionally, when comparing the color difference value L*, it could be noticed that when the LSOPC content rose especially when it reached 4 mg/g, the color difference L* increased visibly. It demonstrated that the most notable effect on the color of the cookies’ skin was the synergistic interaction between LSOPC’s own color and the color of the browning product.

### 3.7 Texture properties

Table 1 provided a summary of the findings. The values of hardness ranged from 1808.606 to 3008.872. The skin hardness of the LSOPC samples (LSOPC-1, LSOPC-2, LSOPC-3, and LSOPC-4) was all reduced when compared to the blank samples (LSOPC-0), despite the absence of a consistent correlation between the additional LSOPC concentration. The texture of the LSOPC-added cookie samples was better and they weren’t as hard in relation to the epidermis.

### 3.8 Flavor

#### 3.8.1 Electronic nose principal component analysis

By recombining the original indicators into a new set of unrelated and comprehensive indicators, principal component analysis (PCA) attempted to replace the original indicators. In order to reflect as much as feasible in accordance with one’s practical needs, some extensive indications could be eliminated. By altering the axis, this analytical technique could distinguish the sample (49). As indicated in Table 2, the PEN3 type electronic nose employed in this work was a metal oxide sensor type with an array of 10 metal oxide gas sensors.

**TABLE 2:** Sensitive substances of PEN3 electronic nose sensor.

Serial number	Sensor name	Performance description
1	W1C	Sensitive to aromatic components, benzene
2	W5S	High sensitivity, sensitive to nitrogen oxides
3	W3C	Sensitive to aromatic ingredients, ammonia sensitive
4	W6S	Mainly sensitive to hydrogen
5	W5C	Sensitive to short chain alkanes, aromatic components
6	W1S	Sensitive to methyl groups
7	W1W	Sensitive to sulfides
8	W2S	Sensitive to alcohols and aldehydes and ketones
9	W2W	Sensitive to aromatic components, organic sulfides
10	W3S	Sensitive to long-chain alkanes

**FIGURE 7:**
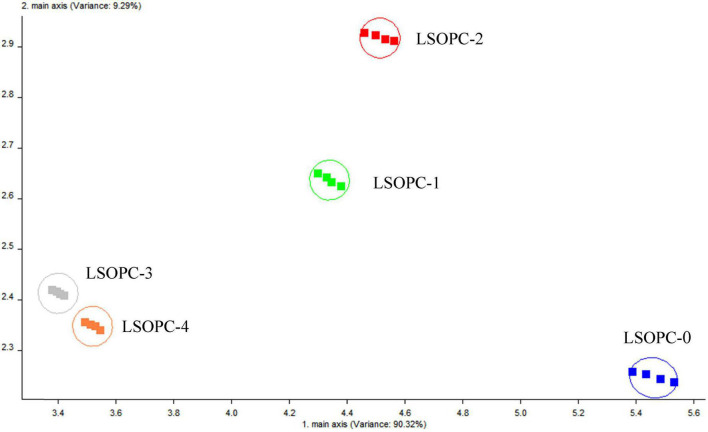
Flavor signals of cookies.

The PCA approach was used to do mathematical statistics on these odor fingerprint data in order to further assess the flavor variations between the five cookie samples. According to Figure 7, the contribution of PC1, the first primary component, was 90.32%, while PC1 and PC2 together provided 99.61%. This indicated that the first major component’s accounted for 90.32% of the odor components detected in the electronic nose. This might be a more accurate representation of the initial data. According to the high-dimensional matrix data, the electronic nose was able to accurately identify odor changes in different cookie samples. Under the identical testing circumstances, the distinction between the LSOPC-containing and LSOPC-free cookie samples were more obvious. Since each group had its own area and did not totally overlap, the groups were unique and had particular study value.

#### 3.8.2 Gas quality measurement

Volatile flavor compounds were further analyzed and identified using GC-MS to determine changes in flavor components with or without LSOPC. The five cookie samples contained a total of three acids, ten ester, one phenolic, three aldehydes, and eleven additional flavoring ingredients, as indicated in Table 3. In the cookie samples without LSOPC, LSOPC-0 recognized 21 flavor components, LSOPC-1, LSOPC-2, LSOPC-3, and LSOPC-4 detected 12, 16, 6, and 10 flavor components, respectively. In the LSOPC-0 group, 4 aldehydes were found; in the LSPOC-1 group, 3 aldehydes were found; in the LSOPC-2 group, 3 aldehydes were found; and in the LSOPC-3 group, 1 aldehyde was found. One aldehyde species and three aldehydes were present in the LSOPC-4 group. Then, the addition of LSOPC led to a minor decrease in aldehyde species, a chemical that served as a precursor for the creation of AGEs (47), which suggested subtly that the reduction of AGEs in cookies increased their quality and safety.

**TABLE 3:** Flavor substances analysis by GC-MS.

Number of categories	Total flavor substance	Aldehydes	Phenols	Esters	Acids	Alkanes	Olefins hydrocarbons
LSOPC-0	21	4	1	8	0	4	0
LSOPC-1	16	3	0	6	1	4	0
LSOPC-2	12	3	0	4	0	3	1
LSOPC-3	6	1	0	3	0	0	0
LSOPC-4	10	3	0	4	0	0	1

### 3.9 Rheological measurement

The rheological properties of cookies dough under dynamic conditions were assessed using the sweep frequency test. The energy recovered per cycle of deformation was calculated using the storage modulus (G’), which could be used to define the solid or elastic properties of dough. Additionally, the loss modulus (G”) was an estimation of the energy released as heat each cycle of deformation, indicating the viscous response of dough (if LSOPC was added to 0.4%, the rheometer sensing range will be surpassed) (50, 51).

**FIGURE 8:**
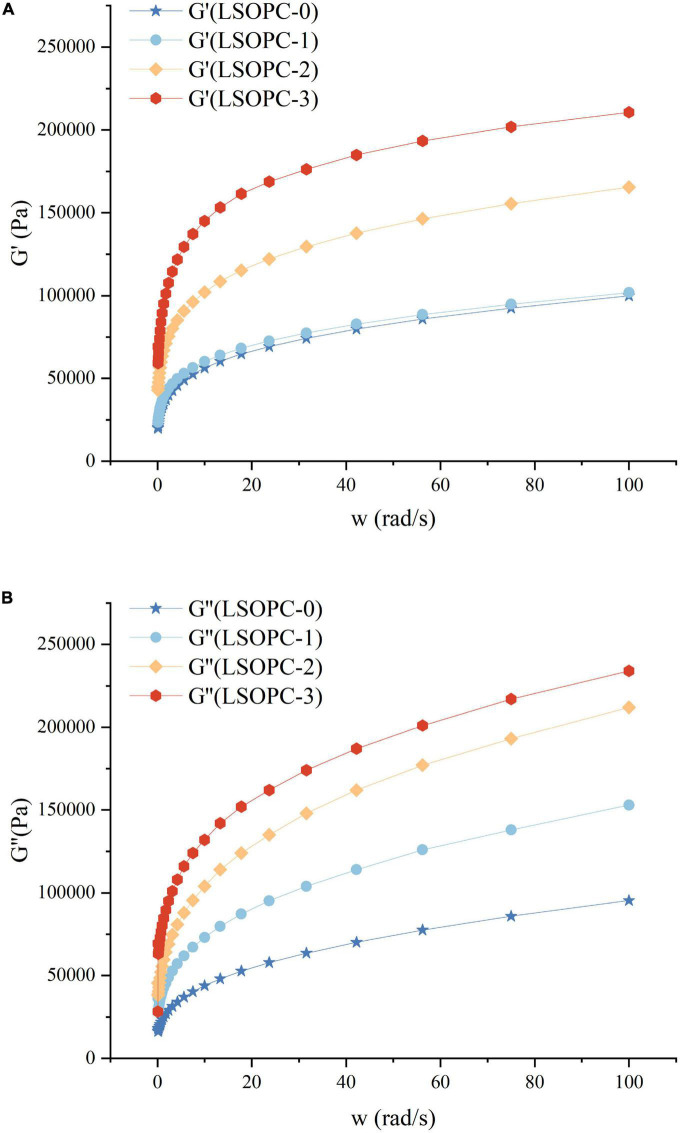
Effect of LSOPC on G’ **(A)** and G” **(B)**.

In Figure 8, it was evident that G’ and G” increased when LSOPC concentration increased, which was noted as a strong frequency dependence. Obviously, G’ was larger than G” throughout the whole frequency range, exhibiting the typical rheological properties of crosslinking polymers (52, 53). The LSOPC formed numerous hydrogen bonds with gluten as a result of the benzene hydroxyl, which increased the physical crosslinking degree of the gluten network and helped to form a consistent “grid” structure (Figure 8). As a result, the G’ and G” of the dough increased and the cohesiveness was improved. Although LSOPC reduced the dough’s tensile strength, its active ingredients helped gluten dough become somewhat more tough and ductile. In particular, LSOPC enhanced gluten’s lamellar structure, which improved tensile distance. Meanwhile, the “grid” structure was created by the interaction of LSOPC with gluten, which increased the dough’s strength and dynamic modulus. The use of LSOPC and its active ingredients in cookies products would be greatly influenced by these findings.

## 4 Conclusion

By using various LSOPC concentrations, the effects of LSOPC on the suppression of AGEs development and sensory quality during cookies baking were examined in this study. In the experimental concentration range, the inhibitory and antioxidant effects of AGEs in cookies rose with increasing concentrations of LSOPC, indicating that LSOPC could successfully prevent the development of AGEs in cookies. As the concentration of LSOPC rose, the sensory quality of the cookies improved as the hue turned gradually brown and the hunger grew. Texture and rheological examination revealed that after the addition of cookies with LSOPC, the cookies became less firm, softer and spongy in the mouth, and had greater dough strength. The findings of the electronic nose PCA analysis revealed that the flavors of cookies with various LSOPC concentrations clearly differed from one another. In conclusion, the addition of LSOPC to cookies enhanced its antioxidant activity, reduced the production of MR harmful chemicals AGEs, and to some extent enhanced its sensory quality. When taken as a whole, this makes it a likely nutrient option for a well-liked meal in the future.

## Data availability statement

The original contributions presented in this study are included in the article/supplementary material, further inquiries can be directed to the corresponding authors.

## Author contributions

QW: writing—review and editing. JT: data curation and writing—original draft. JQ: conceptualization, methodology, investigation, and writing—original draft. ZC: data curation and methodology. BL and JX: data curation. WJ: conceptualization and methodology. NF: supervision and funding acquisition. All authors contributed to the article and approved the submitted version.
